# Evaluation of Preoperative Chemotherapy or Radiation and Overall Survival in Patients With Nonmetastatic, Resectable Retroperitoneal Sarcoma

**DOI:** 10.1001/jamanetworkopen.2020.25529

**Published:** 2020-11-11

**Authors:** Sung Jun Ma, Oluwadamilola T. Oladeru, Mark K. Farrugia, Rohil Shekher, Austin J. Iovoli, Anurag K. Singh

**Affiliations:** 1 Department of Radiation Medicine, Roswell Park Comprehensive Cancer Center, Buffalo, New York; 2 Department of Radiation Oncology, Massachusetts General Hospital, Boston

## Abstract

This cohort study compares overall survival among patients with nonmetastatic, resectable retroperitoneal sarcoma treated with surgical treatment alone vs surgical treatment and chemotherapy or radiation.

## Introduction

Soft tissue sarcoma represents approximately 1% of all cancers, and up to 20% of soft tissue sarcoma occurs in the retroperitoneum.^1^ Locoregional failure occurs in up to 50% of cases.^2^ Although a 2019 prospective trial^3^ suggested no survival benefit with preoperative radiation, the National Comprehensive Cancer Network (NCCN) guidelines on neoadjuvant treatments for nonmetastatic, resectable retroperitoneal sarcoma are heterogeneous and are at the discretion of clinicians.^4^ Given a paucity of large prospective data, clinical benefit of neoadjuvant interventions remains unclear. We performed a retrospective cohort study using a nationwide oncology database to compare surgical treatment alone vs surgical treatment and preoperative therapy regimens.

## Methods

The Roswell Park Comprehensive Cancer Center institutional review board approved this cohort study and determined that informed consent was not required because the database was deidentified and publicly available to those who applied through the American College of Surgeons website. Our study follows the Strengthening the Reporting of Observational Studies in Epidemiology (STROBE) reporting guideline. The National Cancer Database (NCDB) was queried for patients diagnosed between 2006 and 2015 with nonmetastatic, resectable retroperitoneal sarcoma. We searched for individuals treated with surgical procedure alone or surgical procedure following preoperative chemotherapy or radiation. Primary end point was overall survival, evaluated by Kaplan-Meier method, log-rank test, and Cox multivariable analysis. To reduce selection bias, propensity score matching was performed (using treatment facility type, treatment facility volume, and patient age, sex, Charlson/Deyo comorbidity score, income level, insurance type, histological characteristics, tumor grade, year of diagnosis, T and N staging, surgical procedure type, surgical margin, postoperative readmission, and duration of postoperative inpatient admission). To address immortal time bias, individuals who survived less than 6 months after diagnosis were excluded as a conditional landmark (eAppendix in the Supplement). Analyses were performed March 2020 to May 2020 using R statistical software version 3.6.1 (R Project for Statistical Computing). All *P* values were evaluated using 2-sided Cox proportional hazard multivariable analysis, and *P* values less than .05 were considered statistically significant.

## Results

Of 7857 patients who met our inclusion criteria, with median (interquartile range [IQR]) age 63 (53-72) years, 4003 (50.9%) were men; 6814 patients (86.7%) underwent surgical treatment alone, 850 patients (10.8%) had preoperative radiation, and 193 patients (2.5%) received preoperative chemotherapy (Table). The median (IQR) follow-up was 48.7 (27.6-76.8) months. Most patients with preoperative therapies were treated at academic, high-volume facilities and had simple or radical resections with negative margins and a longer postoperative inpatient admission compared with patients with no preoperative therapies (Table). On multivariable analysis adjusted for facility type, age, sex, income, Charlson/Deyo comorbidity score, histological characteristics, tumor grade, tumor size, surgical type, surgical margin, and postoperative inpatient duration, addition of preoperative radiation was associated with improved overall survival (hazard ratio [HR], 0.88; 95% CI, 0.77-0.99; *P* = .03) while the addition of preoperative chemotherapy was associated with lower overall survival (HR, 1.54; 95% CI, 1.27-1.88; *P* < .001). A similar association of improved overall survival was found in patients with preoperative radiation in 844 matched pairs (HR, 0.83; 95% CI, 0.72-0.97; *P* = .02) but not in patients with preoperative chemotherapy in 186 matched pairs (HR, 1.44; 95% CI, 1.07-1.94; *P* = .02) (Figure). Compared with preoperative radiation therapy, preoperative chemotherapy was associated with lower overall survival in 169 matched pairs (HR, 1.58; 95% CI, 1.15-2.18; *P* = .005) (Figure).

**Table.** Baseline Characteristics for Cohorts Before and After Matching

Characteristic	Before matching				After matching^a^								
	Surgical treatment alone, No. (%) (n = 6814)	With radiation therapy, No. (%) (n = 850)	With chemotherapy, No. (%) (n = 193)	*P* value	Surgical treatment alone, No. (%) (n = 844)	With radiation therapy, No. (%) (n = 844)	*P* value	Surgical treatment alone, No. (%) (n = 186)	With chemotherapy, No. (%) (n = 186)	*P* value	With radiation therapy, No. (%) (n = 169)	With chemotherapy, No. (%) (n = 169)	*P* value
Facility type													
Nonacademic	2858 (41.9)	254 (29.9)	43 (22.3)	<.001	253 (30.0)	254 (30.1)	.99	50 (26.9)	42 (22.6)	.45	47 (27.8)	42 (24.9)	.82
Academic	3478 (51.0)	540 (63.5)	121 (62.7)		537 (63.6)	535 (63.4)		106 (57.0)	118 (63.4)		104 (61.5)	108 (63.9)	
Not available	478 (7.0)	56 (6.6)	29 (15.0)		54 (6.4)	55 (6.5)		30 (16.1)	26 (14.0)		18 (10.7)	19 (11.2)	
Facility volume													
Low	387 (5.7)	34 (4.0)	6 (3.1)	<.001	27 (3.2)	34 (4.0)	.68	5 (2.7)	6 (3.2)	>.99	4 (2.4)	6 (3.6)	.55
Intermediate	1053 (15.5)	80 (9.4)	18 (9.3)		81 (9.6)	80 (9.5)		19 (10.2)	18 (9.7)		22 (13.0)	16 (9.5)	
High	5374 (78.9)	736 (86.6)	169 (87.6)		736 (87.2)	730 (86.5)		162 (87.1)	162 (87.1)		143 (84.6)	147 (87.0)	
Age, y													
<65	3679 (54.0)	475 (55.9)	144 (74.6)	<.001	477 (56.5)	470 (55.7)	.77	134 (72.0)	138 (74.2)	.73	117 (69.2)	122 (72.2)	.63
≥65	3135 (46.0)	375 (44.1)	49 (25.4)		367 (43.5)	374 (44.3)		52 (28.0)	48 (25.8)		52 (30.8)	47 (27.8)	
Sex													
Women	3371 (49.5)	380 (44.7)	103 (53.4)	.02	378 (44.8)	377 (44.7)	>.99	98 (52.7)	101 (54.3)	.84	96 (56.8)	87 (51.5)	.38
Men	3443 (50.5)	470 (55.3)	90 (46.6)		466 (55.2)	467 (55.3)		88 (47.3)	85 (45.7)		73 (43.2)	82 (48.5)	
Charlson/Deyo comorbidity score													
0	5208 (76.4)	669 (78.7)	166 (86.0)	.01	654 (77.5)	664 (78.7)	.81	163 (87.6)	159 (85.5)	.90	138 (81.7)	142 (84.0)	.90
1	1224 (18.0)	137 (16.1)	23 (11.9)		141 (16.7)	136 (16.1)		19 (10.2)	23 (12.4)		27 (16.0)	23 (13.6)	
≥2	382 (5.6)	44 (5.2)	4 (2.1)		49 (5.8)	44 (5.2)		4 (2.2)	4 (2.2)		4 (2.4)	4 (2.4)	
Income level													
≥Median	4189 (61.5)	504 (59.3)	109 (56.5)	.20	527 (62.4)	502 (59.5)	.35	102 (54.8)	106 (57.0)	.94	91 (53.8)	92 (54.4)	>.99
<Median	2521 (37.0)	331 (38.9)	78 (40.4)		299 (35.4)	327 (38.7)		79 (42.5)	75 (40.3)		72 (42.6)	71 (42.0)	
Not available	104 (1.5)	15 (1.8)	6 (3.1)		18 (2.1)	15 (1.8)		5 (2.7)	5 (2.7)		6 (3.6)	6 (3.6)	
Insurance type													
Uninsured	210 (3.1)	29 (3.4)	6 (3.1)	<.001	30 (3.6)	28 (3.3)	.99	7 (3.8)	6 (3.2)	.35	6 (3.6)	6 (3.6)	.80
Private	3159 (46.4)	397 (46.7)	104 (53.9)		397 (47.0)	392 (46.4)		89 (47.8)	102 (54.8)		90 (53.3)	97 (57.4)	
Government	3243 (47.6)	409 (48.1)	66 (34.2)		402 (47.6)	409 (48.5)		67 (36.0)	64 (34.4)		64 (37.9)	60 (35.5)	
Not available	202 (3.0)	15 (1.8)	17 (8.8)		15 (1.8)	15 (1.8)		23 (12.4)	14 (7.5)		9 (5.3)	6 (3.6)	
Histological characteristics													
Leiomyosarcoma	1794 (26.3)	231 (27.2)	69 (35.8)	<.001	224 (26.5)	230 (27.3)	.94	66 (35.5)	68 (36.6)	.97	65 (38.5)	61 (36.1)	.97
Sarcoma, NOS	315 (4.6)	55 (6.5)	13 (6.7)		62 (7.3)	54 (6.4)		9 (4.8)	13 (7.0)		9 (5.3)	11 (6.5)	
Spindle cell sarcoma	183 (2.7)	35 (4.1)	8 (4.1)		44 (5.2)	35 (4.1)		8 (4.3)	7 (3.8)		7 (4.1)	6 (3.6)	
Giant cell sarcoma	166 (2.4)	83 (9.8)	20 (10.4)		81 (9.6)	80 (9.5)		17 (9.1)	16 (8.6)		24 (14.2)	19 (11.2)	
Fibrosarcoma	74 (1.1)	8 (0.9)	1 (0.5)		8 (0.9)	8 (0.9)		1 (0.5)	1 (0.5)		0 (0.0)	1 (0.6)	
Malignant fibrous histiocytoma	162 (2.4)	23 (2.7)	5 (2.6)		18 (2.1)	23 (2.7)		7 (3.8)	5 (2.7)		8 (4.7)	5 (3.0)	
Low-grade liposarcoma	2380 (34.9)	154 (18.1)	8 (4.1)		146 (17.3)	154 (18.2)		4 (2.2)	8 (4.3)		6 (3.6)	8 (4.7)	
Intermediate-grade liposarcoma	304 (4.5)	60 (7.1)	12 (6.2)		54 (6.4)	60 (7.1)		15 (8.1)	12 (6.5)		8 (4.7)	11 (6.5)	
High-grade liposarcoma	1307 (19.2)	184 (21.6)	46 (23.8)		185 (21.9)	184 (21.8)		46 (24.7)	46 (24.7)		40 (23.7)	44 (26.0)	
Hemangiosarcoma	66 (1.0)	3 (0.4)	10 (5.2)		6 (0.7)	3 (0.4)		11 (5.9)	9 (4.8)		1 (0.6)	2 (1.2)	
Malignant peripheral nerve sheath tumor	63 (0.9)	14 (1.6)	1 (0.5)		16 (1.9)	13 (1.5)		2 (1.1)	1 (0.5)		1 (0.6)	1 (0.6)	
Tumor grade													
Well differentiated	2585 (37.9)	166 (19.5)	14 (7.3)	<.001	170 (20.1)	166 (19.7)	>.99	16 (8.6)	14 (7.5)	.81	11 (6.5)	14 (8.3)	.83
Moderately differentiated	956 (14.0)	119 (14.0)	16 (8.3)		118 (14.0)	119 (14.1)		14 (7.5)	16 (8.6)		20 (11.8)	15 (8.9)	
Poorly differentiated	1361 (20.0)	233 (27.4)	64 (33.2)		228 (27.0)	229 (27.1)		70 (37.6)	60 (32.3)		54 (32.0)	56 (33.1)	
Others	837 (12.3)	160 (18.8)	55 (28.5)		158 (18.7)	159 (18.8)		47 (25.3)	53 (28.5)		43 (25.4)	47 (27.8)	
Not available	1075 (15.8)	172 (20.2)	44 (22.8)		170 (20.1)	171 (20.3)		39 (21.0)	43 (23.1)		41 (24.3)	37 (21.9)	
Year of diagnosis													
2006-2010	3113 (45.7)	336 (39.5)	82 (42.5)	.002	340 (40.3)	333 (39.5)	.77	80 (43.0)	79 (42.5)	>.99	69 (40.8)	73 (43.2)	.74
2011-2015	3701 (54.3)	514 (60.5)	111 (57.5)		504 (59.7)	511 (60.5)		106 (57.0)	107 (57.5)		100 (59.2)	96 (56.8)	
T staging													
1	1051 (15.4)	66 (7.8)	14 (7.3)	<.001	73 (8.6)	66 (7.8)	.93	11 (5.9)	14 (7.5)	.79	17 (10.1)	12 (7.1)	.72
2	1488 (21.8)	235 (27.6)	41 (21.2)		239 (28.3)	233 (27.6)		45 (24.2)	39 (21.0)		39 (23.1)	37 (21.9)	
3	1148 (16.8)	190 (22.4)	38 (19.7)		176 (20.9)	186 (22.0)		44 (23.7)	38 (20.4)		34 (20.1)	34 (20.1)	
4	2710 (39.8)	330 (38.8)	85 (44.0)		330 (39.1)	330 (39.1)		74 (39.8)	81 (43.5)		74 (43.8)	77 (45.6)	
Not available	417 (6.1)	29 (3.4)	15 (7.8)		26 (3.1)	29 (3.4)		12 (6.5)	14 (7.5)		5 (3.0)	9 (5.3)	
N staging													
0	4893 (71.8)	700 (82.4)	142 (73.6)	<.001	685 (81.2)	696 (82.5)	.79	126 (67.7)	138 (74.2)	.40	125 (74.0)	126 (74.6)	>.99
1	70 (1.0)	28 (3.3)	6 (3.1)		28 (3.3)	26 (3.1)		4 (2.2)	4 (2.2)		6 (3.6)	6 (3.6)	
Not available	1851 (27.2)	122 (14.4)	45 (23.3)		131 (15.5)	122 (14.5)		56 (30.1)	44 (23.7)		38 (22.5)	37 (21.9)	
Surgical procedure													
Local excision	2356 (34.6)	153 (18.0)	28 (14.5)	<.001	155 (18.4)	152 (18.0)	.70	36 (19.4)	28 (15.1)	.58	19 (11.2)	24 (14.2)	.80
Simple resection	3144 (46.1)	463 (54.5)	115 (59.6)		435 (51.5)	458 (54.3)		104 (55.9)	109 (58.6)		97 (57.4)	98 (58.0)	
Radical resection	975 (14.3)	195 (22.9)	35 (18.1)		212 (25.1)	195 (23.1)		28 (15.1)	34 (18.3)		36 (21.3)	33 (19.5)	
Not available	339 (5.0)	39 (4.6)	15 (7.8)		42 (5.0)	39 (4.6)		18 (9.7)	15 (8.1)		17 (10.1)	14 (8.3)	
Surgical margin													
Negative	4015 (58.9)	569 (66.9)	123 (63.7)	<.001	552 (65.4)	568 (67.3)	.68	117 (62.9)	119 (64.0)	.66	105 (62.1)	109 (64.5)	.88
Positive	1759 (25.8)	215 (25.3)	41 (21.2)		226 (26.8)	210 (24.9)		35 (18.8)	39 (21.0)		44 (26.0)	40 (23.7)	
Not available	1040 (15.3)	66 (7.8)	29 (15.0)		66 (7.8)	66 (7.8)		34 (18.3)	28 (15.1)		20 (11.8)	20 (11.8)	
Readmission within 30 d													
None	6337 (93.0)	772 (90.8)	176 (91.2)	.28	774 (91.7)	766 (90.8)	.84	170 (91.4)	170 (91.4)	.71	160 (94.7)	154 (91.1)	.56
Unplanned	279 (4.1)	50 (5.9)	9 (4.7)		44 (5.2)	50 (5.9)		10 (5.4)	8 (4.3)		3 (1.8)	7 (4.1)	
Planned	91 (1.3)	12 (1.4)	3 (1.6)		9 (1.1)	12 (1.4)		3 (1.6)	3 (1.6)		2 (1.2)	3 (1.8)	
Others	5 (0.1)	0 (0.0)	0 (0.0)		1 (0.1)	0 (0.0)		1 (0.5)	0 (0.0)		0 (0.0)	0 (0.0)	
Not available	102 (1.5)	16 (1.9)	5 (2.6)		16 (1.9)	16 (1.9)		2 (1.1)	5 (2.7)		4 (2.4)	5 (3.0)	
Postoperative inpatient duration, d													
<6	2665 (39.1)	225 (26.5)	45 (23.3)	<.001	222 (26.3)	224 (26.5)	.99	46 (24.7)	42 (22.6)	.41	38 (22.5)	37 (21.9)	.97
≥6	3473 (51.0)	514 (60.5)	118 (61.1)		510 (60.4)	510 (60.4)		104 (55.9)	116 (62.4)		106 (62.7)	108 (63.9)	
Not available	676 (9.9)	111 (13.1)	30 (15.5)		112 (13.3)	110 (13.0)		36 (19.4)	28 (15.1)		25 (14.8)	24 (14.2)	

Abbreviation: NOS, not otherwise specified.

^a^ Three different matched pairs were performed (ie, surgical treatment alone vs with radiation, surgical treatment alone vs with chemotherapy, and with radiation vs with chemotherapy). Total number (n value) of each treatment cohort within each matched pair is the same.

**Figure.**
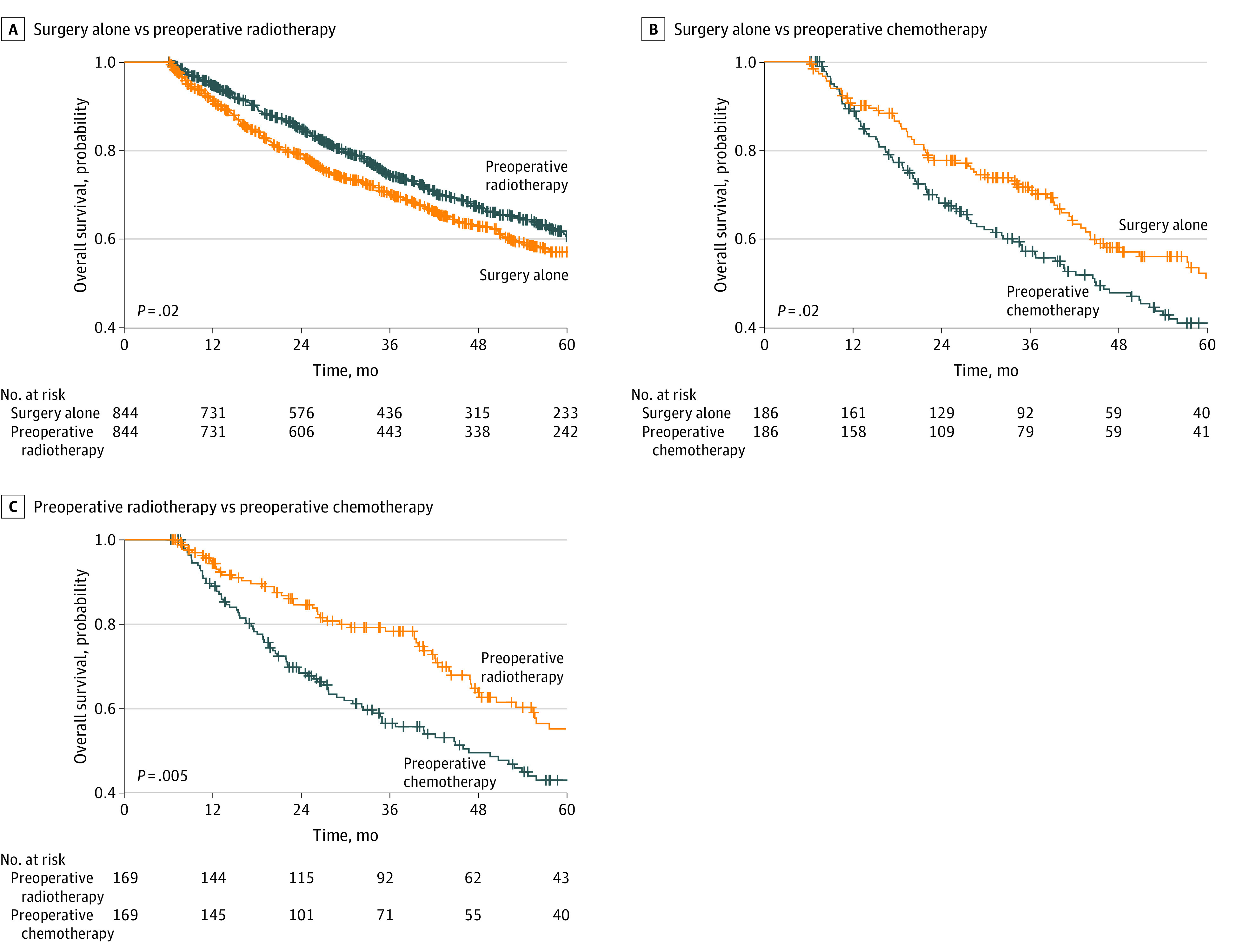
Kaplan-Meier Survival Curves After Matching

## Discussion

To our knowledge, this cohort study is the first study to use a national registry database to report the comparison of survival outcomes among patients receiving surgical treatment alone, preoperative chemotherapy, and preoperative radiation therapy for retroperitoneal sarcoma. Our finding of overall survival benefits from preoperative radiation therapy is consistent with a 2016 retrospective study^5^ and the current NCCN guideline recommendation. However, our finding is inconsistent with a 2019 prospective trial^3^ that did not show overall survival benefit with radiation therapy, in part due to smaller sample sizes and shorter follow-up periods, with reporting outcomes at 3 years. In addition, worse survival outcomes seen in preoperative chemotherapy may be due to mortality secondary to locoregional failure.^2^

This study has several limitations. Some pertinent factors, including performance status, were not captured in the NCDB, and unmeasured confounding may be present despite matching. However, postoperative readmissions and duration of postoperative inpatient admission were matched as proxy measures for postoperative complications and performance status after patients completed treatments.^6^ Given the small sample size of preoperative therapy subgroups, our findings may not be generalizable to other patient populations. While we await further prospective trials, such as a randomized phase III study of neoadjuvant chemotherapy followed by surgery vs surgery alone for patients with high-risk retroperitoneal sarcoma (NCT04031677), our study may inform clinicians’ decisions concerning preoperative therapies in patients with resectable retroperitoneal sarcoma.

## Notes

Supplement. **eAppendix.** Supplementary Methods
