# Supplementary material for: Evaluation of Preoperative Chemotherapy or Radiation and Overall Survival in Patients With Nonmetastatic, Resectable Retroperitoneal Sarcoma
Source: JAMA Netw Open. 2020 Nov 11;3(11):e2025529. doi: 10.1001/jamanetworkopen.2020.25529 (PMC7658730; doi:10.1001/jamanetworkopen.2020.25529)
Supplement: Supplement. — eAppendix. Supplementary Methods [file jamanetwopen-e2025529-s001.pdf]

Supplemental Online Content

Ma SJ, Oladeru OT, Farrugia MK, Shekher R, Iovoli AJ, Singh AK. Evaluation of preoperative chemotherapy or radiation and overall survival in patients with nonmetastatic, resectable retroperitoneal sarcoma. *JAMA Netw Open*. 2020;3(11):e2025529. doi:10.1001/jamanetworkopen.2020.25529

eAppendix. Supplementary Methods

This supplemental material has been provided by the authors to give readers additional information about their work.

© 2020 Ma SJ et al. *JAMA Network Open.*

eAppendix. Supplementary Methods

Institutional review board at Roswell Park Comprehensive Cancer Center approved our study (BDR-131220). Our study follows the Strengthening the Reporting of Observational Studies in Epidemiology (STROBE) reporting guideline.

All missing values were defined as unknown for our analysis. Pertinent variables such as medical comorbidities, performance status, type and duration of chemotherapy, toxicity profiles, tumor recurrence events, and cancer specific mortality were not captured in the NCDB. The primary endpoint was overall survival (OS) defined as the time duration between diagnosis and the last follow-up or death.

Treatment groups were divided into 3 cohorts: surgery alone, preoperative radiation therapy followed by surgery, and preoperative chemotherapy followed by surgery. Follow up was until the end of 2017. Those who survived less than 6 months after their diagnoses were excluded for analysis based on the estimated number of weeks from the diagnosis to the completion of treatments: up to 4 weeks for coordinating consultation with multiple oncology disciplines followed by multidisciplinary discussions after the biopsy, 5-6 weeks of radiation, up to 6-8 weeks between the completion of radiation and the surgery, and additional 4-8 weeks of postoperative management as indicated.

Categorical and continuous variables were compared using Fisher exact test and Mann-Whitney U test, respectively. Cox proportional hazard multivariable analysis (MVA) model was built based on all statistically significant variables from the Cox univariable analysis followed by a backward stepwise elimination. Variables of interest for analysis include facility type, facility volume, age, gender, comorbidity burden, income, insurance, histology, tumor grade, year of diagnosis, T and N staging, treatment regimens, postoperative readmission, and postoperative inpatient duration. Assumptions of Cox proportional hazards model were verified based on Schoenfeld residual method.

When propensity score matching was performed, variables of interest were those from the final Cox MVA model and other clinically relevant factors as shown in the Table 1. The nearest neighbor method was used in a 1:1 ratio without a replacement. A caliper distance of 0.1 of the standard deviation of the logit of the propensity score was used. The standardized difference of all variables were lower than 0.1, suggestive of adequate match.

All p values were two-sided and p values less than 0.05 were considered statistically significant.

© 2020 Ma SJ et al. *JAMA Network Open.*
